# Mechanical, Fracture, and Microstructural Assessment of Carbon-Fiber-Reinforced Geopolymer Composites Containing Na_2_O

**DOI:** 10.3390/polym13213852

**Published:** 2021-11-08

**Authors:** Ahmad Rashedi, Riadh Marzouki, Ali Raza, Nurul Fazita Mohammad Rawi, J. Naveen

**Affiliations:** 1 College of Engineering, IT & Environment, Charles Darwin University, Ellengowan Drive, Casuarina, NT 0810, Australia; 2 Department of Chemistry, College of Science, King Khalid University, Abha 61413, Saudi Arabia; rmarzouki@kku.edu.sa; 3 Department of Chemistry, Faculty of Sciences of Sfax, University of Sfax, Sfax 3029, Tunisia; 4 Department of Civil Engineering, University of Engineering and Technology Taxila, Taxila 47050, Pakistan; ali.raza@uettaxila.edu.pk; 5 Bioresource Technology Division, School of Industrial Technology, Universiti Sains Malaysia, George Town 11800, Malaysia; fazita@usm.my; 6 School of Mechanical Engineering, Vellore Institute of Technology, Vellore 632014, India; gandhi.naveen66@gmail.com

**Keywords:** carbon fibers, geopolymer, nano-sodium dioxide, compressive strength, hardness, scanning electron microscopy

## Abstract

For a sustainable environment, geopolymer (GPO) paste can be used in the construction industry instead of Portland cement. Nowadays, sustainable construction and high-efficacy composites are demanding. Therefore, in the present investigation, the mechanical and microstructural efficacy of carbon-fiber-reinforced fly ash-based GPO with different percentages of nano-sodium dioxide (NS) were studied. The investigated percentages of NS were 0%, 1%, 2%, 3%, and 4%. For all the samples, the carbon fiber content was kept the same at 0.5% by weight. Different percentages of NS for all five fabricated GPO composite pastes were assessed with scanning electron microscopy (SEM). Various mechanical parameters of GPO—the compressive strength, toughness modulus, hardness, toughness indices, impact strength, fracture toughness, flexural strength, and elastic modulus—were evaluated. The results revealed that the use of 3% NS was the most effective for ameliorating the mechanical, microstructural, and fracture behavior of GPO. The use of 3% NS in carbon-fiber-reinforced GPO paste showed the maximum improvements of 22%, 46%, 30%, 40%, 14%, 38.4%, 50.2%, 31%, and 64% for the compressive strength, flexural strength, elastic modulus, toughness modulus, hardness, compressive stiffness, bending stiffness, fracture toughness, and impact strength, respectively. The SEM study showed that the inclusion of NS improved the microstructure and delivered a denser GPO paste by improving the interfacial zones and quickening the polymerization reaction.

## 1. Introduction

Studies on the geopolymer (GPO) composites are in the spotlight of advanced research due to the high requirements for sustainable construction and high-efficacy composites [1,2]. Environmental pollution is increasing due to the high carbon footprint by-products of Portland cement production and the emission of 5% CO_2_ globally [3]. Due to outstanding efficacy in the construction pastes, geopolymers can be used instead of Portland cement [4,5,6,7]. By activating aluminosilicate compounds (fly ash by means of an alkali), these composites behave like binders [8]. The durability and mechanical efficacy of GPO are more or equal to the normal strength of plain concrete [9,10,11]. To use them in construction, other modern techniques should be explored. The early strength of GPO is reduced when prepared with fly ash, and it has limited use because of its brittle nature when the requirement is of flexural strength (FLS) capacity is high. Hence, other modern techniques should be explored to tackle this problem.

Much research has been conducted on the normal strength parameter of both plain concrete and GPO, and it has been revealed that when added to GPO, carbon fibers can significantly improve its mechanical properties [5,12,13,14,15,16,17]. Carbon fibers have the ability to transfer the stresses in GPO paste, resulting in a delay of crack generation [5,12,16]. The literature has shown that the mechanical efficacy of GPO with carbon fibers is greater and the mechanical efficacy of control GPO is lesser without fibers [12,13,14,15]. The compressive strength (COS) of GPO paste (metakaolin-based) with a carbon fiber length of 24 mm is more than that with a carbon fiber length of 12 mm [5]. Longer setting times, stronger COS, and reductions in dry shrinkage were observed after adding carbon fibers instead of fly ash to GPO composites [16]. When 10 wt% carbon fibers were added, a 37% increase in the COS of GPO pasted was observed in fly ash-based GPO [14]. There was no significant improvement in the COS of GPO paste after the inclusion of 15–30 wt.% carbon fibers. So, in the production of GPO composites, carbon fibers can be added as reinforcement.

Research on nanomaterial has been conducted to improve construction composites [18,19,20,21]. It was uncovered from examinations that the durability and mechanical performance of both GPO composites and cementitious can be improved with low amounts of nano-materials (rather than miniature materials). With their superfine size, nanomaterials can improve the qualities of composite materials. Because of their performance as nucleation destinations and nanofillers, nanomaterials lead to a speed increase in the polymerization response. To explore the impacts of nanomaterials on the ordinary strength of plain concrete, some lab work has been conducted [22,23]. There is very little literature available on the inclusion of nanomaterial in geopolymers. Past studies of the impacts nanomaterials on construction composites used nano-sodium dioxide, nano-silica [24,25], or nano-titanium oxide [26,27]. Recent research on 3% nano-sodium dioxide (NS) was conducted by Assaedi et al. [21] without adding carbon fibers; in this work, it was uncovered that the addition of up to 2% of NS led to improvements in the mechanical properties of GPO mixes, and when the amount of NS was increased to 3%, there was a decrease in the mechanical properties of the GPO mix. Nevertheless, the consolidated utilization of NS and carbon fiber has not been contemplated in GPO mixes, and jointly using them in industry development is essential. In this manner, the current experiment was conducted to observe the mechanical performance of carbon-fiber-reinforced geopolymer mixes with different percentages of NS.

In our current experimental work, we set up samples of GPO paste with a fixed carbon fiber amount of 0.5% and the addition of different percentages of NS (0%, 1%, 2%, 3%, and 4% by weight). We observed the effects of various percentages on the mechanical properties of GPO composite pastes, including COS, compressive stress–strain curves, FLS, toughness indices, bending stress–strain curves, impact strength (IMS), elastic modulus, toughness modulus, fracture toughness (FRT), and hardness (HAD). The microstructure effect of NS on the GPO pastes was also studied by means of scanning electron microscopy (SEM). In the development of better fly ash-based GPO composite pastes in the construction industry, the current study will aid understanding and encourage the use of carbon fibers and NS for the improvement of the mechanical and microstructural efficacy of GPO.

## 2. Experimental Setup

### 2.1. Materials and Methods

**Figure 1:**
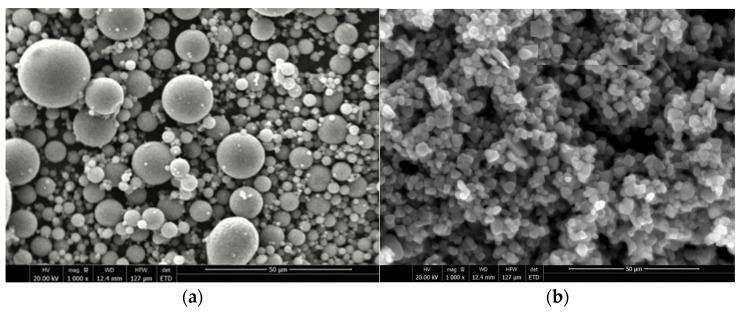
SEM micrographs of (**a**) fly ash and (**b**) NS.

Table 1 shows the chemical composition of the class F fly ash used in current study as an aluminosilicate precursor, tested according to ASTM-C618 [28]. The class C fly ash non-cement mortar demonstrated a higher strength compared to class F fly ash at ambient temperature. In contrast, the class F fly ash non-cement mortars exhibited better efficacy than class C when cured at high temperatures. Moreover, class F fly ash is generally more effective than class C fly ash in reducing the heat of hydration. In the present study, the GPO was synthesized at 80 degrees Celsius; therefore, class F fly ash was used in this investigation. Figure 1a shows an SEM analysis of fly ash. For the preparation of GPO composite pastes, binary activator sodium silicate and sodium hydroxide were utilized. Sodium hydroxide purity was 98%, and it was used in form of flakes; the solution form of sodium silicate was used. Sodium silicate comprised 29.4% SiO_2_, 14.7% Na_2_O, and 55.9% H_2_O.

The different percentages of NS in the GPO composite pastes were studied with the characteristics mentioned in Table 2. The average particle size of the white crystalline powdered NS was 20 nm. Figure 1b shows the SEM micrographs of NS. In our current work, the tensile strength of carbon fibers was 4000 MPa, and the modulus of elasticity was 242 GPa. The carbon fiber length was 12 mm. Table 3 shows the properties of the carbon fibers used in the present study.

Different amounts of NS with FA in the GPO composite mix were ready and tried for COS, FRT, elastic modulus, FLS, HAD, toughness modulus, and SEM. The proportion or alkali to fly ash was fixed at 0.45. For all GPO composite blends, the proportion of sodium silicate to sodium hydroxide was fixed at 2.5. Pellets were broken up in refined water and attained a 10 M concentration of sodium hydroxide. Furthermore, they were left untouched for 24 h prior to blending in with the GPO mix. Different concentrations (1%, 2%, 3%, and 4%) of NS with 0.5% of carbon fibers supported the GPO composite mix. Table 4 shows the composition of the different GPO mixes.

### 2.2. Sample Preparation and Curing

**Figure 2:**
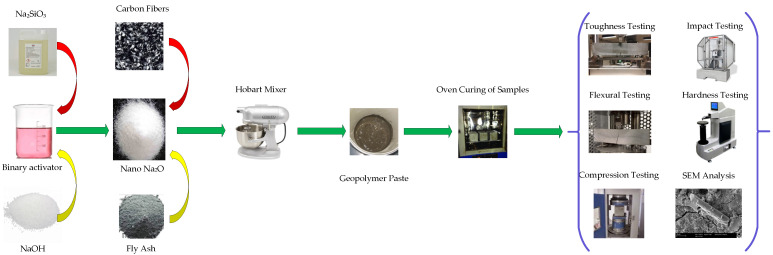
Construction of GPO nanocomposites.

To mix the GPO paste, we used a Hobart mixer at a 500 rev./min unrest speed. At low speed, the carbon fiber and fly ash were dry-blended for 5 min. The high-speed blending of different concentrations of NS was finished with an alkali arrangement, and it was stopped following the obtained of a homogenous GPO blend. Molds with the new mix were saved for 24 h on a stove at temperature of 80 °C. The curing process was conducted for the following 28 days at room temperature following demolding to increase strength. Figure 2 shows the abovementioned procedure.

### 2.3. Testing Procedures of Samples

#### 2.3.1. COS Test

The total number of cube samples tested according to ASTM D 695 [29] was 30. The dimensions of the cubes was 20 mm × 20 mm × 20 mm. All samples were prepared at predefined percentages, i.e., 0%, 1%, 2%, 3%, and 4% NS. COS was assessed for all mixes, and the average results are reported for each one. Based on the stress–strain graph, we divided the ultimate load by the cross-sectional area of the sample to obtain the COS.

#### 2.3.2. FLS Test

As with COS, the total number samples tested according to ASTM C–293 [30] was 30. The size of the FLS samples was 60 mm × 20 mm × 20 mm. All the samples were manufactured according to predefined concentrations of 0%, 1%, 2%, 3%, and 4% NS. The standard system used for testing (three-point bending tests) is shown below. FLS was found for every one of the five blends, and the average results are reported. To ensure the adequacy of this three-point flexural test, the pace of loading was kept at 1 mm/min. Based on the bending stress–strain charts, we obtained definitive bending loading values used them to estimate the FLS ($\sigma_{F}$) with the following formula:

(1) $$\Sigma_{F}=\frac{3}{2}\frac{P_{m}S}{BW^{2}}$$

where $P_{m}$ = ultimate bending load at crack extension; S = sample span; B = sample width; and W = sample thickness or depth.

#### 2.3.3. FRT Test

As with COS and FLS, the total number of samples tested was 30. The dimensions for the FRT samples were 70 mm × 20 mm × 20 mm. All the samples were prepared with predefined percentages of NS, i.e., 0%, 1%, 2%, 3%, and 4%. The standard procedure adopted for testing (FRT) is provided below. During this FRT test, the rate of loading was kept the same at 0.5 mm/min; FRT was found for all mixes and reported as the average results of each one. Initially, a crack was observed with a length to thickness ratio of 0.4. Equation (2) was used for the calculation of this FRT.

(2) $$K_{IC}=\frac{p_{m}S}{BW^{\frac{3}{2}}}f\left(\frac{a}{W}\right)$$

where $K_{IC}$ = FRT; $p_{m}$ = ultimate bending load at the crack; S = sample span; B = sample width; $f\left(\frac{a}{W}\right)$ = coefficient that was calculated by means of Equation (3) [31]; a = initial crack length; and W is = initial crack thickness.

(3) $$f\left(\frac{a}{W}\right)=\frac{3{\left(a/W\right)}^{1/2}\left[1.99-\left(a/W\right)\left(1-a/W\right)\times \left(2.15-3.93a/W+2.7a^{2}/W^{2}\right)\right]}{2\left(1+2a/W\right){\left(1-a/W\right)}^{3/2}}$$

#### 2.3.4. HAD Test

The HAD of all samples was assessed with a Rockwell H scale by means of the Rockwell HAD tester. The standard adopted for this procedure was ASTM D785 [32]. All the samples were prepared with NS concentrations of 0%, 1%, 2%, 3%, and 4%. This test was conducted for all blends at room temperatures, and we report the average outcomes. To ensure a high exactness, the sample surfaces were cleaned.

#### 2.3.5. IMS Test

This test was performed for all samples by means of a ‘Zwick Charpy’ impact tester with a one Joule pendulum hammer. The length of the samples was 40 mm. Equation (4) was used for the calculation of IMS ($\sigma_{i}$) [21]:

(4) σi=EA

where E = impact energy required to break a sample with a ligament of area A.

#### 2.3.6. Microstructural Analysis through SEM

The examination of fractured GPO composite pastes including surface morphology and failure mechanism was conducted with the help of scanning electron microscopy (SEM). TO ensure testing adequacy, the sample was placed in a vacuum desiccator for 48 hr. After this, it was stuck to an aluminum stub. The samples were covered with gold to avoid the creation of electron charge. A magnifying instrument was used to observe the break development and crack surface morphology of blends. Damage was observed, and its reasons were examined.

## 3. Discussion of Results

### 3.1. Compressive Strength

**Figure 3:**
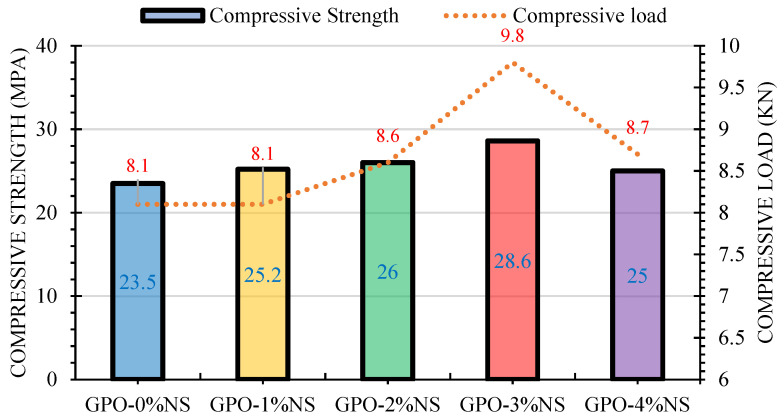
Effect of different quantities of NS on the COS and compressive loads of mixes.

Figure 3 shows the COS of GPO composite pastes with different NS percentages (0%, 1%, 2%, 3%, and 4%) and with a fixed carbon fiber percentage of 0.5%. It can be seen that COS was improved by 3% NS. A huge in-wrinkle could be seen with the addition of 3% NS, and the COS was diminished with the addition of 4% NS in comparison to the control blend. Compared to GPO without NS, and GPO with 1% NS (GPO-1%NS) presented a 7% enhancement in COS. There was an 11% improvement in the COS with the addition of 2% NS (GPO-2%NS) in the GPO composite mix due to the fact that small amount of NS acted as filler, sealing the small openings between carbon fibers and GPO paste. There was an improvement of 22% in the COS of GPO composite blends following the addition of 3% NS with a fixed carbon fiber concentration of 0.5%. The improvement caused by the addition of 3% NS was caused by the ability of the nanomaterials (3% NS) to impede the formation of nanovoids and microvoids, as well as the capacity of NS to speed up the polymerization response process. Additionally, there was a 6% enhancement in the COS following the addition of 4% NS (GPO-4%NS) in the GPO composite mix. The addition of NS sped up the polymerization response interaction due to its ability to act as a nucleation locale [20,33,34]. Thus, to obtain good results for this test, 3% NS was selected.

**Figure 4:**
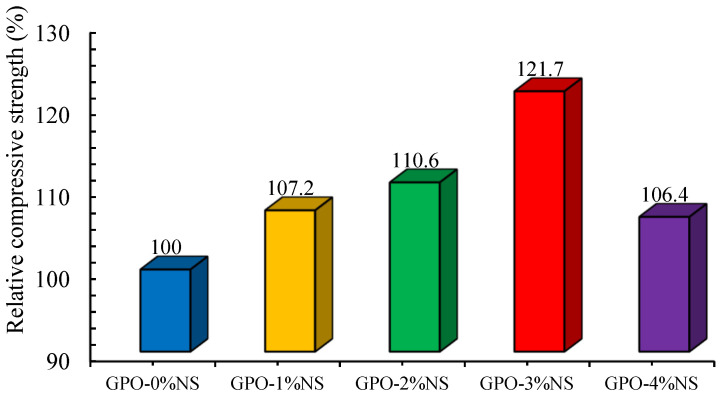
Relative COS of carbon-fiber-reinforced GPO composite pastes with various contents of NS.

The COS with carbon fibers in most GPO composite pastes with different percentages of NS (0%, 1%, 2%, 3%, and 4%) was greater than that of pastes without carbon fibers [21]. This proves the viability of adding 0.5% of carbon fiber to a GPO mix with different concentrations of NS. Following the incorporation of carbon fibers, an improvement was seen due to the more densified microstructure and equivalent higher load-conveying limit of the mix, as displayed in Figure 3. Along these lines, it was clear that the addition of 0.5% carbon fiber and 4% NS improved the COS of the blends. The correlation of the relative COS GPO composite mixes and without NS and various concentrations of NS (such as the control blend (GPO-0%NS)) is displayed in Figure 4. The COS of GPO composite blends with NS concentrations of 1%, 2%, 3%, and 4% and a carbon fiber concentration of 0.5% was increased by 107.2%, 110.6%, 121.7%, and 106.4%, respectively, in comparison to the COS of the control blend with 0.5% of carbon fiber and no NS.

### 3.2. Compressive Stress–Strain Behavior

**Figure 5:**
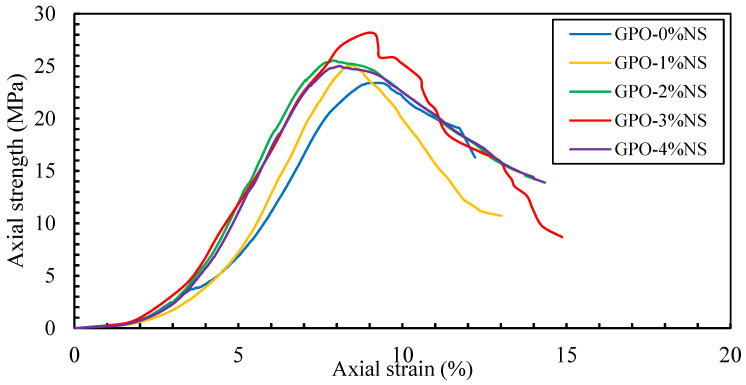
Compressive stress–strain efficacy of carbon-fiber-reinforced GPO composite paste with various contents of NS.

**Figure 6:**
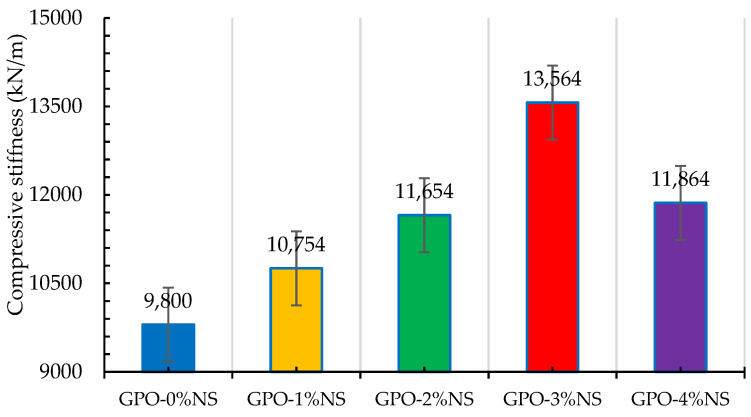
Effect of different quantities of NS on the compressive stiffness of GPO pastes.

Figure 5 shows the axial compressive stress–strain performance of GPO composites with 0.5% of carbon fiber and 0%, 1%, 2%, 3% and 4% of NS. The control sample (GPO-0%NS) showed less stiffness than the samples with different concentrations of NS, showing that the incorporation of NS increased the compressive stress, generally hardening the GPO composites and allowed them to bear higher compressive loading at lower compressive strains, as seen in Figure 6. There were improvements in axial strength upon the addition of NS at lower strains, as can be seen in the compressive stress–strain curves. These were caused by the improved interaction of the polymerization response and denser GPO mix. Adding NS accelerated the course of polymerization response because of its capacity as nucleation destinations for beneficial items inside the GPO composites [20,33,34], thus leading to higher stiffness. Additionally, samples at lower compressive strains showed higher COS degradation when various concentrations of NS were added. This may likewise be credited to the higher stiffness of samples before the post-peak stage that caused the breaking of the GPO mix in the post-stacking area of the stress–strain curves due to the weaker conductance of the mixes with NS. The compressive stiffness values of GPO-1%NS, GPO-2%NS, GPO-3%NS, and GPO-4%NS were 9.7%, 18.9%, 38.4, and 21.1%, respectively, values that were greater than that of GPO-0%NS (no NS). In this way, GPO-3%NS presented the best compressive stiffness of 13,564 kN/m.

**Figure 7:**
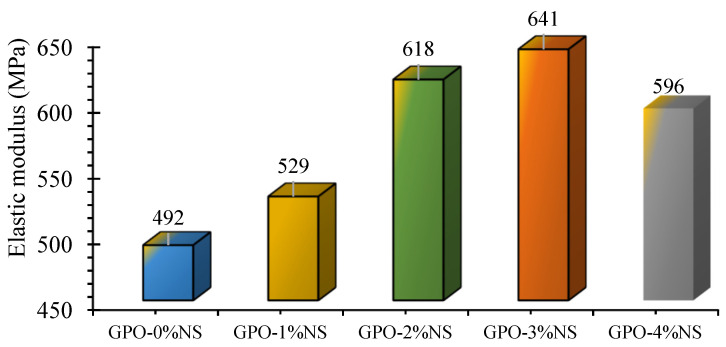
Elastic moduli of carbon-fiber-reinforced GPO composite pastes with various contents of NS.

The samples showed higher stress–strain curve slopes following the addition of various contents of NS due to the greater stiffness of the GPO mix. Figure 7 shows that the elastic moduli of these mix were higher than that of the control mix (GPO-0%NS). The elastic moduli GPO-1%NS, GPO-2%NS, and GPO-3%NS were 8%, 26%, 30%, and 21%, respectively, and these values were greater than the GPO-0%NS (no NS). Accordingly, GPO-3%NS presented the most elevated elastic modulus (641 MPa) and best axial stiffness. This significant advancement in the modulus of GPO may be associated with the enhancement of the polymerization reaction caused by the incorporation of NS.

### 3.3. Flexural Strength

**Figure 8:**
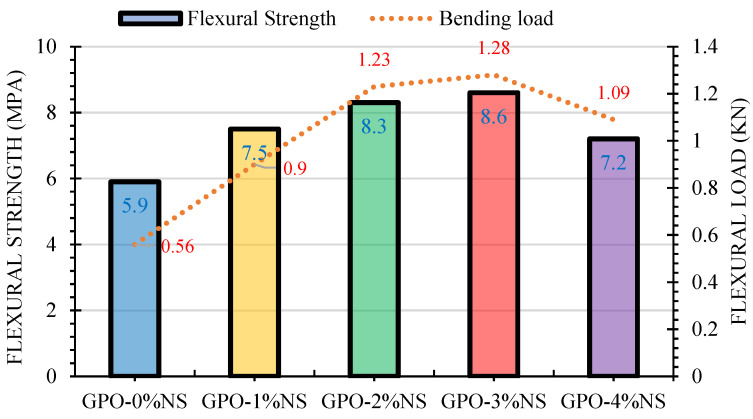
Effect of different quantities of NS on the FLS and bending loads of mixes.

The FLS test results of GPO composite pastes with different contents of NS and 0.5% of carbon fiber are shown in Figure 8. This FLS test was conducted when these materials were presented to resist a load. The average estimations of six samples from every GPO composite blend showed that the FLS of the mix intensified at up to 3% of NS before a decrease in the FLS began. All GPO composite mixes with NS had better FLS values than the 0% NS composite. This improved strength might have been caused by the speeding up or accelerating of the polymerization response process, prompting the densification of the composite blend and the improvement of the microstructure with the NS particles. Moreover, Figure 8 shows that following the addition of NS, the samples were able to acquire a higher bowing burden because of the increment in the stiffness of the mix.

**Figure 9:**
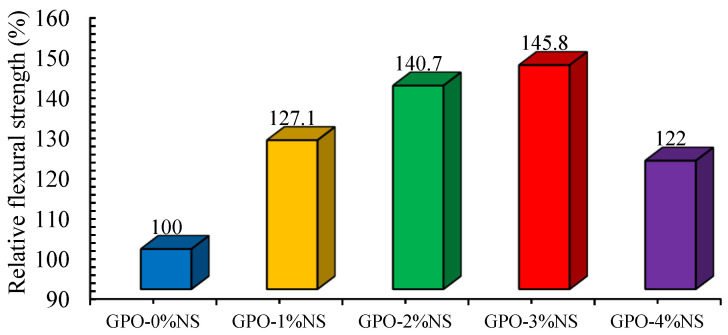
Comparison of the FLS of carbon-fiber-reinforced GPO composite pastes with different amounts of NS.

The addition of more than 3% NS decreased the FLS of GPO composite mixes compared to the other GPO mixes with NS. This impact could be ascribed to the accumulation of nanoparticles. Nevertheless, it is clear from the results shown in Figure 3 that the COS values were different at various concentrations of nanoparticles. To further examine the effects of the nanoparticles, we proposed an assortment of top-to-bottom experimentations. Unreinforced polymer mixes with the addition of 3% of nano-particles were already studied, and comparable discoveries were made in [21]. Here, it was observed that the addition of 1–2% of NS without carbon fiber further decreased the FLS. In contrast, the addition of 0.5% of carbon fiber and 1%, 2%, 3%, and 4% of NS resulted in increases in FLS in comparison to the control mix (GPO-0%NS). The FLS for GPO paste was found to be optimum with 3% of NS. In accordance with the experimentations by Alomayri [35], it was found that the addition of 1% and 2% of nano-alumina to geopolymer composites enhanced the FLS by 5.6% and 44.5%, respectively. As such, the utilization of NS with carbon fiber has a more noteworthy impact on FLS than when only using nano-alumina. The results found with and without NS at various concentrations are displayed in Figure 9. After adding 1%, 2%, 3%, and 4% of NS alongside carbon fiber, the FLS increased up to 127.1%, 140.7%, 145.8%, and 122%, respectively.

### 3.4. Bending Stress–Strain Behavior

**Figure 10:**
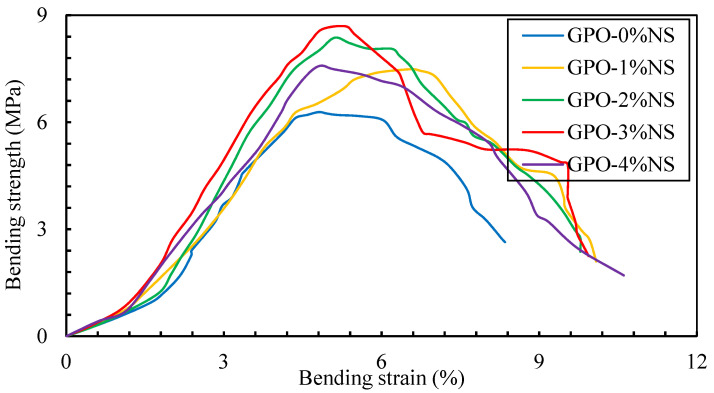
Variations in the bending stress behavior of fiber-reinforced pastes with different amounts of NS.

**Figure 11:**
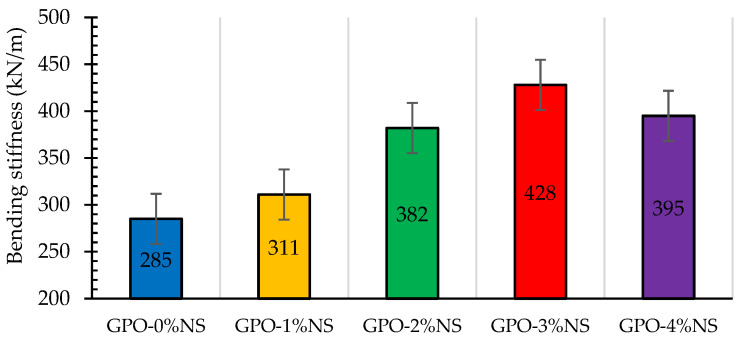
Impact of different amounts of NS on GPO paste stiffness.

The bending stress–strain behavior results of geopolymers with different amounts of NS along and 0.5% of carbon fibers are shown in Figure 10. A sample without NS showed a lesser bending load and little stiffness, while the addition of various amounts of NS upgraded the bending stress in comparison to the control sample (GPO-0%NS). As such, it can be inferred that the incorporation of various concentrations of NS yielded a stiffer GPO mix that was able to bear higher loads at higher strains. The greatest stiffness was found by adding 3% of NS, thus provided the best toughness modulus. The plotted curves for bending stress and strains showed that an increased amount of NS increased the bending loads at lower strains. This expansion in bending loads was because of the arrangement of a denser mix and the speeding up of the advancement of polymerization response via the presence of NS, which acted as nucleation sites and led to higher bending stiffness values [20,33,34]. Simultaneously, various concentrations of NS diminished the bowing strength at higher strain stages because the samples were more hardened before the post-top phase, which led to breaks at that stage due the weakness of the GPO mix without NS. The bending stiffness values of GPO-1%NS, GPO-2%NS, GPO-3%NS, and GPO-4%NS were 9.1%, 34%, 50.2, and 38.6%, respectively, and these values were better than that of GPO-0%NS. Accordingly, GPO-3%NS showed the most elevated stiffness of 428 kN/m. The bending stiffness values of all examined samples are shown in Figure 11.

**Figure 12:**
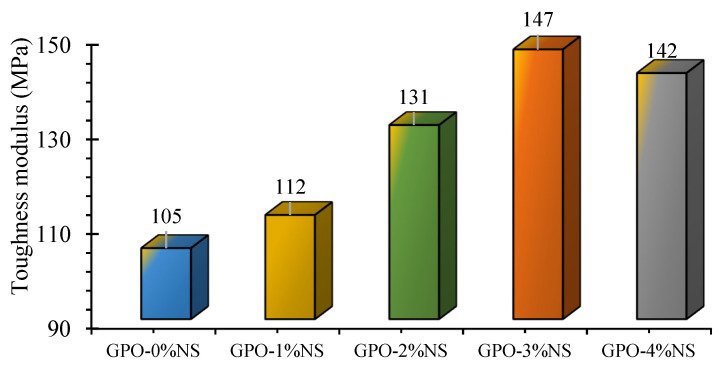
Variation in the modulus of toughness in carbon-fiber-reinforced GPO pastes due to different proportions of NS.

The moduli of toughness for different GPO pastes were studied; the results are shown in Figure 12. The samples yielded more extreme stress–strain curve slopes with 3% of NS. At the end of the day, samples with NS showed modulus of toughness values than the control blend. The toughness modulus of the GPO mix kept increasing up to 3% NS, before diminishing, though it remained higher than that of the control sample. The addition of 1% NS alongside 0.5% of fiber carbon enhanced stiffness by 7% in contrast to the control blend (GPO-0%NS). Likewise, 2% of NS increased the strength by 25% compared to the control mix. The modulus of strength with 3% NS was 40% better than that of the control blend. Tests of mixes with 3% NS yielded 147 MPa of bending stiffness, which was viewed as ideal for any remaining nanomaterials with 3% NS. Likewise, the modulus of toughness with 4% of NS was 35% better than that of the control mix. This increase in strength modulus was caused by the mix becoming denser with the speeding-up of polymerization response following the incorporation of NS [20,34].

### 3.5. Flexural Load–Deflection Curves and Toughness Indices

**Figure 13:**
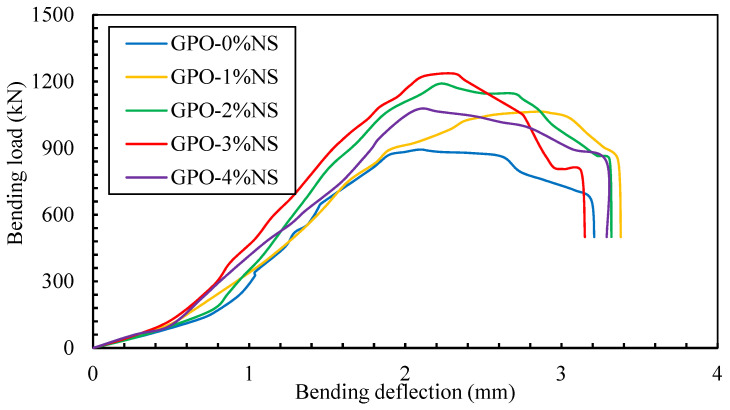
Load–mid span deflection curves of carbon-fiber-reinforced GPO samples.

The deflection behavior of bending load applied at mid-span for carbon-fiber-reinforced geopolymers composite pastes with different proportions of NS is shown in Figure 13. When nanoparticles were added, the definitive loading of GPO was diminished. It was additionally uncovered that the deflection of the GPO mix with NS particles was higher than that of the control sample. This expansion of the curve was caused the presence of nanoparticles that hindered the proliferation of break development. Carbon fibers refracted from the breaks at the interface between the GPO lattice and fibers. It was observed during the bending test that geopolymers with a small proportion of NS were highly deflected and showed fractures that could not be recovered. As the bending load increased, the bending of nanocomposites was found to be the elastic range and recognizable redirection was displayed. At the point when the load surpassed the elastic range, plastic deformity occurred. As this stage was crossed, the applied load was gradually decreased. This made a long tail that resulted in higher FRT. The diversion of the GPO framework with 1% NS was higher than at all other concentrations. 

The displacement improvement could be ascribed to a decent fiber–lattice bond, which improved the capacity of these mixes by catching energy. This opened up convoluted pathways for the break spread, which helped to enhanced the bending strength. It is shown in the load–deflection curve that the presence of NS improved the mechanical performance of the GPO mix. Likewise, 3% NS improved the FRT of the carbon-fiber-reinforced GPO mix. Additionally, more than 3% of NS lessened redirection. The results showed that the presence of NS led to deflection improvements because of the arrangement of a fiber–lattice bond, but it additionally decreased the diversion of all models because of the creation of a brittle composite. The consolidation of NS fortified the connection between the fiber and GPO lattice, but it also diminished the energy absorption process due to the draw-out and debonding peculiarities of the fibers. Due to powerless interfacial attachment, the course of inception and spread increased, thus prompting longer fiber take-out lengths. Unexpectedly, fiber cracks were more grounded than the draw-out fibers in the structure. Increased the NS from 3% to 4% decreased the mid-span deflection.

**Figure 14:**
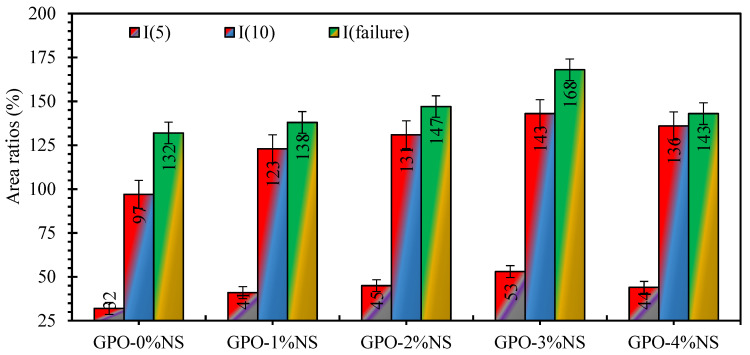
Toughness indices ($I_{5}$, $I_{10}$, and $I_{failure}$) for all carbon-fiber-reinforced GPO samples.

The guidelines provided by ASTM C1018 were utilized to calculate the flexural toughness indices of carbon-fiber-reinforced GPO samples. Three different toughness indices—$I_{5}$, $I_{10}$, and $I_{failure}$—were calculated to examine the flexural toughness. Index $I_{5}$ is defined as the ratio between three times the area of the bending load–deflection curve up to the deflection at the first crack and the area of the curve at the first crack. Similarly, Index $I_{10}$ is defined as the ratio between 5.5 times the area of bending load–deflection curve up to the deflection at the first crack and the area of the curve at the first crack. Finally, $I_{failure}$ is the ratio of the area of the complete bending load–deflection curve in proportion to the area of the curve up to the deflection at the first crack. The tensile behavior of pure GPO composites was weaker than that of those with NS, and they were brittle under loading. Hence, their toughness values were smaller, so fiber reinforcement can always be recommended [21]. Toughness indices values ($I_{5}$, $I_{10}$, and $I_{failure}$) are shown in Figure 14 for all the carbon-fiber-reinforced GPO samples. The toughness indices of the carbon-fiber-reinforced GPO sample with 1% NS were increased by 28.1%, 26.8%, and 4.5% for $I_{5}$, $I_{10}$, and $I_{failure}$, respectively, in comparison to the control samples. Similarly, 2% NS incorporation enhanced the toughness indices by 40.6%, 35.1%, and 11.4% for $I_{5}$, $I_{10}$, and $I_{failure}$, respectively, in comparison to the control samples. Similarly, 3% NS incorporation enhanced the toughness indices by 65.6%, 47.4%, and 27.3% for $I_{5}$, $I_{10}$, and $I_{failure}$, respectively, in comparison to the control samples. The incorporation of 4% NS enhanced the toughness indices by 37.5%, 40.2%, and 8.3% for $I_{5}$, $I_{10}$, and $I_{failure}$, respectively, compared to the control mix. It can be concluded that toughness indices of the GPO mixes were greatly improved by the incorporation of NS due to the that strong bond developed between the fibers and GPO pastes due to the percentage of NS and a high proportion of GPO gel. The incorporation of 3% of NS provided an optimum toughness index value.

### 3.6. Fracture Toughness

**Figure 15:**
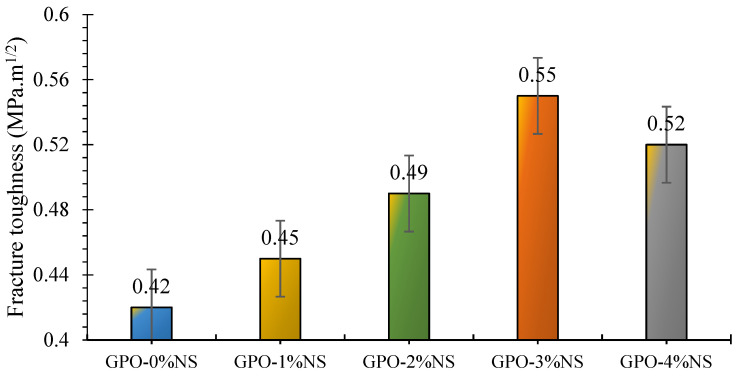
FRT of carbon-fiber-reinforced GPO composite pastes with various contents of NS.

To counter the spread of crack widths, composite materials must have fracture toughness. Figure 15 shows that GPO mixes with NS showed more prominent improvements of FRT than the control mix (GPO-0%NS). Moreover, the FRT values were more than 0.40 MPa·m^1/2^ for all models. The greater improvements of FRT might have been caused by the existence of carbon fibers, which led to solid network arrangement and increased the FRT in the composites. This additionally contributed to changing the inner stresses and prompted enhancements in obstruction against breaks and their proliferation.

With the increase in NS content from 1% to 3%, the FRT of pastes increased, reaching 0.55 MPa·m^1/2^ at 3% NS. Regardless, the FRT of the GPO composites containing NS was greater than that of the control mixes, thus demonstrating that the NS improved the toughness of the GPO mixes. The FRT of GPO-1%NS was increased by 7% in comparison to the control sample (GPO-0%NS). Similarly, the toughness of GPO-2%NS was 17% greater than that of GPO-0%NS. The addition of 3% NS increased the FRT by 31% compared to the control blend; this was the best result. The improvements in the FRT of the GPO mixes may have been caused by the incorporation of NS. The FRT of GPO-4%NS was increased by 24% in comparison to GPO-0%NS. The nanoparticles inside the mix improved the strength of the connect between the GPO mix and fibers, thus leading to a denser mix that hindered breaking. This was because the nanoparticles sped up and refined the polymerization response by enhancing the connection between the composite material and carbon fibers.

### 3.7. Hardness

**Figure 16:**
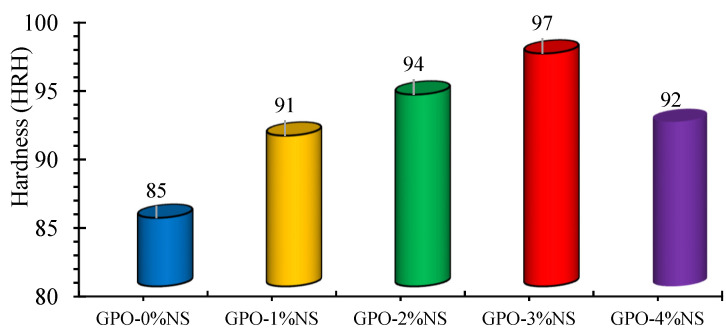
Variation in the HAD values of carbon-fiber-reinforced GPO composite pastes due to different proportions of NS.

The ability to bear localized plastic deformation is referred to as the HAD of the GPO composite paste. Figure 16 shows the impacts of adding 0%, 1%, 2%, 3%, and 4% of NS on the HAD of the mix of carbon-fiber-reinforced geopolymers. We found that the HAD of the GPO mix was improved after adding 1–4% NS. GPO-1%NS showed an increase of 7% in HAD in comparison to the control model, while an improvement of 11% in the HAD of the mix was seen with GPO-2%NS. GPO-3%NS yielded a 14% increase in the HAD. The incorporation of 4% NS decreased the improvement of the HAD to 8% in contrast to the control blend. At different concentrations of NS, the enhancement in HAD was caused by the greater strength of NS particles, which led to stronger interfacial zones between the GPO network and nanoparticles [36]. Finally, the nanoparticles grew nearer, thus causing increased mix stiffness and eventually leading to higher HAD values. The seemingly contradictory impacts of the lattice on distortion and break development were improved by the nanoparticles, which dispersed all through the network by settling over the space between chains of GPO composites. Alomayri [35] found that geopolymers mixes without any kind of fiber and nanoparticle fusion presented 80 HRH worth of HAD. In this paper, HAD values higher than 80 HRH may have been caused by the joining of carbon fibers in mix, thus demonstrating the positive outcomes of carbon fiber incorporation.

### 3.8. Impact Strength

**Figure 17:**
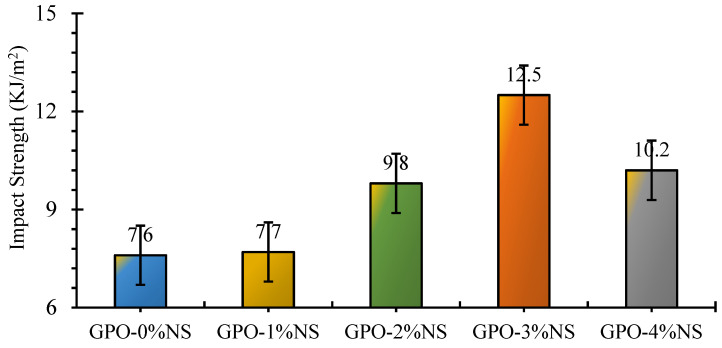
Variation in IMS of carbon-fiber-reinforced GPO composite paste with different proportions of NS.

Figure 17 shows the IMS of numerous carbon-fiber-reinforced GPO composite pastes with various amounts of NS. The results showed that by adding NS particles, the IMS of the GPO composite mixes was enhanced. The incorporation of up to 3% NS led to strength improvements; above this percentage, the strength improvements decreased but remained better than that of the control sample. The IMS of GPO-1%NS was generally similar to that of GPO-0%NS. GPO-2%NS, and GPO-3%NS presented 29% and 64% improved IMS values, respectively, in comparison to GPO-0%NS. Additionally, GPO-4%NS showed an improvement of 34% compared to the control mix. Due to the presence of nanoparticles the mix began decaying in the first stage, thus creating breaks. The nanoparticles inside the composites increased the bond strength between the framework and supporting fibers, which led to denser and stronger structures [37]. During load transfer from the composite matrix to the NS, the interfacial HAD was foundational to the function of the NS nanoparticles. Furthermore, the increase in the impact strength of the carbon-fiber-reinforced GPO composite paste with various amounts of NS was caused by bridging characteristics of the two variables, i.e., NS and carbon fibers.

### 3.9. Microstructural Assessment

**Figure 18:**
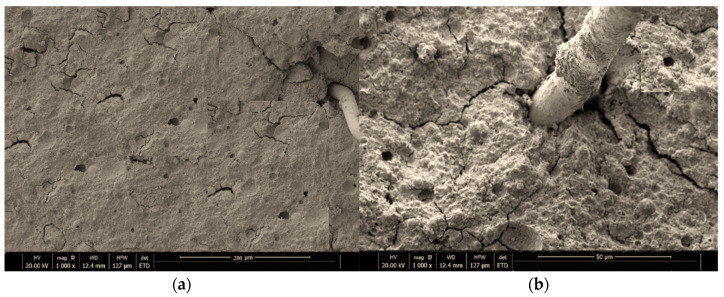

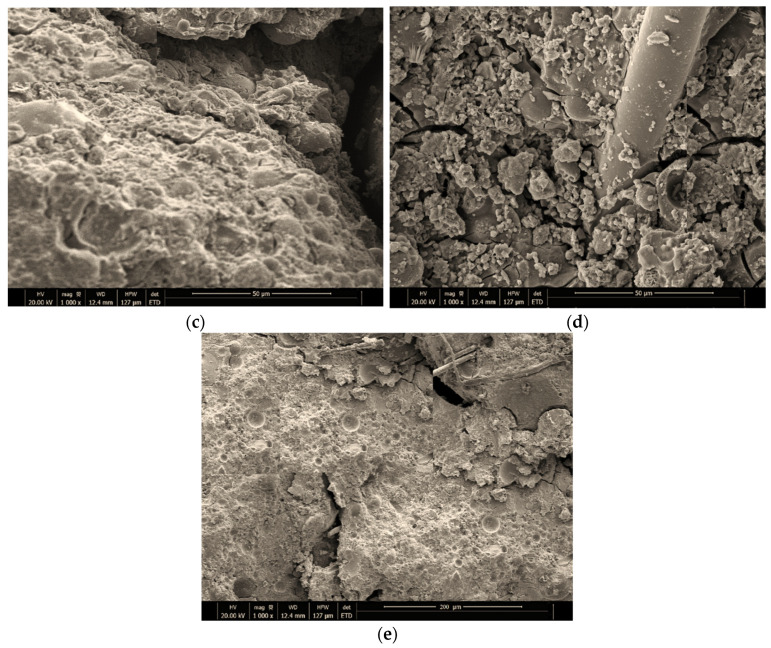
SEM micrographs displaying the microstructures of (**a**) GPO-0%NS, (**b**) GPO-1%NS, (**c**) GPO-2%NS, (**d**) GPO-3%NS, and (**e**) GPO-4%NS.

After performing fracture toughness tests, SEM micrographs were utilized to study the microstructures of GPO composite pastes with different amounts of NS. The SEM assessments of models containing 0%, 1%, 2%, 3%, and 4% of NS are displayed in Figure 18. The significant polymerization response shown by SEM micrographs was caused by the reaction of unreacted fly ash with NS. This response led to the development of a C–S–H gel, which led to the formation of more united and just as dense microstructure as it spread over the pore space [21,38,39]. Moreover, the addition of NS led a denser fastener network after filling the voids between fly ash particles. The breaks diminished the thickness of the mixes as the amount of NS increased. The NS nanoparticles improved the load-moving phenomenon, thus hindering the formation of breaks. This time, NS led to the formation of bonds due the GPO hydration process, which aided in the halting of the initiation of nano-breaks. The SEM micrograph also shows holding among fibers and GPO structure, which might be related to the capacities of NS that refined and increased the density of the GPO mix microstructures.

## 4. Summary and Conclusions

The authors of the present work strived to examine and improve the mechanical, fracture, and microstructural behavior of carbon-fiber-reinforced GPO pastes by adding different amounts of NS. Samples of carbon-fiber-reinforced GPO paste with and without NS were prepared and assessed for the following parameters: COS, compressive stress–strain response, FLS, bending stress–strain response, elastic modulus, toughness indices, FRT, toughness modulus, HAD, and IMS. We present the following observations:

(1) We obtained the maximum COS value of carbon-fiber-reinforced GPO pastes, 22% in comparison to the GPO paste without NS, with 3% NS. This improvement may have resulted from the blocking of nanovoids and microvoids caused by the inclusion of NS, as well as the increased speed of the polymerization reaction.
(2) At 3% NS, the FLS was 46% higher than that of the control blend, possibly because of the expedient impact of the polymerization response cycle and the refinement of the microstructure with the NS particles. This may have prompted the densification of the composite lattice. At 3% NS, we recorded the best improvements of toughness.
(3) At 3% NS, the FRT was raised by 31% in comparison to the control sample, possibly due to the filling property of the nanoparticles and the existence of carbon fibers, as a which moved the interior loads in the lattice and prompted improvements in protection against break initiation.
(4) Following the addition of NS, the IMS of the pastes improved (with 3% NS showing the maximum value of 64% greater than that of the control mix), possibly due to nanoparticles within the GPO pastes. These nanoparticles increased the bond strength between the reinforcing fibers and matrix, and they aided in cracking resistance via the creation of a solid and dense matrix.
(5) The SEM micrographs showed that the appearance of denser structure following the addition of NS, which filled nanovoids between fly ash particles. The SEM micrographs also revealed strong holding between the GPO and carbon fibers, possibly because of the refinement and densification of the composite mixes caused by NS. Therefore, for sustainable development in the construction industry, the mechanical efficacy of carbon-fiber-reinforced GPO composite pastes can be enhanced through NS.

## Figures and Tables

**Table 1:** Chemical composition of fly ash.

Compound	Composition (%)	Compound	Composition (%)
SiO_2_	62.5	Na_2_O	0.82
Al_2_O_3_	27.2	MgO	0.64
CaO	2.56	SO_3_	0.12
Fe_2_O_3_	3.03	P_2_O_5_	0.11
K_2_O	1.81	LOI	1.42

**Table 2:** Properties of NS.

Property	Value	Property	Value
pH	12.0	Appearance	White crystalline powder
Average size	20 nm	Type	Crystalline
Purity	99.9 %	Density	2.28 g/cm^3^

**Table 3:** Physical and chemical characteristics of carbon fibers.

Compound/Feature	Quantity	Compound/Feature	Quantity
Tensile strength	4000 MPa	Elastic modulus	242 GPa
Nominal length	12 mm	Nominal diameter	7 μm
Density	1.81 g/cc	Aspect ratio	1715

**Table 4:** Composition of all five mixes.

Geopolymer Mix	GPO-0%NS	GPO-1%NS	GPO-2%NS	GPO-3%NS	GPO-4%NS
Sodium hydroxide (kg)	0.23	0.23	0.23	0.23	0.23
Fly ash (kg)	1.8	1.8	1.8	1.8	1.8
Micro carbon fibers (wt.%)	0.5	0.5	0.5	0.5	0.5
Sodium silicate (kg)	0.58	0.58	0.58	0.58	0.58
NS (kg)	0	0.01	0.02	0.03	0.04
NS (wt.%)	0	1.0	2.0	3.0	4.0

## Data Availability

The research data are available from the corresponding author.
